# Modified treatment protocol for pediatric systemic lupus erythematosus-associated hemophagocytic lymphohistiocytosis with central nervous system involvement: a case report

**DOI:** 10.3389/fimmu.2026.1796119

**Published:** 2026-06-09

**Authors:** Mingda Tian, Yinzhu Zhang, Danyang Zhang, Chunyi Zhang, Yang Zheng, Junjin Huang, Hebing Zhou, Jiayue Qin, Ming Yang, Yan Yue

**Affiliations:** 1 Department of Pediatrics, Beijing United Family Women’s and Children’s Hospital, Beijing, China; 2 Department of Hematology, Beijing Lu He Hospital, Beijing, China; 3 Department of Medical Affairs, Acornmed Biotechnology Co., Ltd., Beijing, China

**Keywords:** case report, CNS, emapalumab, HLH, SLE

## Abstract

Hemophagocytic lymphohistiocytosis (HLH), a severe, life-threatening hyperinflammatory syndrome driven by dysregulated immune activation, is characterized by rapid clinical deterioration and poor outcomes that pose critical challenges for clinical management. Here, we report on a female patient aged 10 years and 3 months who was diagnosed with systemic lupus erythematosus (SLE)-associated hemophagocytic lymphohistiocytosis with central nervous system (CNS) involvement. Treatment with the conventional HLH-94/2004 regimen failed to control disease progression. Subsequently, second-line salvage therapy, consisting of emapalumab in combination with teniposide and dexamethasone, was initiated to account for the CNS involvement. The patient achieved sustained, complete clinical response following use of this modified therapeutic approach. Our findings suggest that this combination regimen may offer a potentially feasible therapeutic alternative for managing patients with SLE-associated HLH accompanied by CNS involvement; however, given the single-case nature of this report, further validation in larger cohorts is needed.

## Introduction

Hemophagocytic lymphohistiocytosis (HLH) is a severe and life-threatening disease caused by immune dysregulation. Common clinical manifestations of the disease include nonspecific symptoms such as high fever, pancytopenia or oligocythemia, hepatosplenomegaly, coagulation dysfunction, convulsions, and multiple organ failure. According to its triggering factors, HLH can be classified into two major forms: primary and secondary (1). The disease progresses rapidly, and patients experience a median survival time of 2 to 3 months without treatment (2). Primary HLH (pHLH) arises from genetic mutations, whereas secondary HLH (sHLH) is induced by triggers including infections, malignancies, rheumatic disorders. In both forms, central nervous system (CNS) involvement indicates severe disease. Differentiating the attribution of CNS impairment to HLH, macrophage activation syndrome (MAS) or both remains clinically challenging, particularly for patients with MAS, limiting the accuracy of diagnosis and the development of optimal management strategies. Moreover, existing treatment regimens yield relatively unsatisfactory survival outcomes; hence, novel and more potent and targeted interventions are needed.

## Case description

A Mongolian girl aged 10 years and 3 months presented to our institution with a 3-week history of fever, a rash on the face and neck, and leukopenia during a trip to Thailand. She was admitted to the Beijing United Family Women’s and Children’s Hospital health care unit on January 15, 2025.

Upon admission, a physical examination revealed a scattered rash on the upper chest, back, and especially face, where it was predominantly distributed across the cheeks, tip of the nose, and region above the supraorbital ridges. The skin around the nose exhibited bluish–purple discoloration but no blisters or ulcers. Initially, the patient received high-dose glucocorticoid therapy. Although she presented with an obvious rash, given the use of high-dose glucocorticoids, we assessed the levels of complement C3 and C4, antinuclear antibody (ANA), double-stranded DNA (dsDNA), lupus markers, and other relevant indicators. All measured parameters were within the normal limits, except complement C3, with a concentration of 0.08 g/L; moreover, no renal impairment or other typical SLE-related manifestations were observed. The patient did not fully meet the diagnostic criteria for autoimmune rheumatic diseases, particularly SLE. Moreover, screening for infectious and malignant etiologies was negative. Genetic testing via whole-exome sequencing was negative, and blood metagenomic next-generation sequencing (NGS) did not reveal any pathogens. The results of HLH-related index assessments met the HLH-2004 criteria (3); these findings included fever ≥ 38.5°C persisting for more than 7 days, cytopenia (white blood cell (WBC) count 1.2 × 10^9^/L, hemoglobin (Hb) 81 g/L, platelet (PLT) count 70 × 10^9^/L), elevated liver enzymes (alanine aminotransferase (ALT) 254 IU/L, aspartate aminotransferase (AST) 1008 IU/L), an enlarged liver, spleen, and lymph nodes, elevated ferritin (2450 μg/L) and soluble CD25 (sCD25; 2367 U/L), decreased NK cell activity (12.71%) and abnormalities in coagulation function with decreased fibrinogen (102 mg/dL). The bone marrow was infiltrated with HLH cells. Cytokine profiling revealed significantly elevated levels of interleukin (IL)-1β, IL-2, IL-4, IL-5, IL-6, IL-8, IL-10, IFN-γ, and tumor necrosis factor alpha (TNF-α). Moreover, the patient’s triglyceride, fibrinogen, and sCD25 levels approached the thresholds specified in the diagnostic criteria (see **Tables 1**, **2**).

**Table 1:** Patient characteristics at diagnosis.

HLH-2004 criteria	Case findings
Fever ≥ 38.5°C	Tmax: 39°C
Splenomegaly	Positive
Peripheral blood cytopenia with at least two of the following:	
Absolute neutrophile count Hemoglobin Platelet count < 100,000/μL	0.6 × 10^9^/L 78 g/L 62 × 10^9^/L
Hypertriglyceridemia (fasting triglycerides > 265 mg/dL)	280 mg/dL
Hypofibrinogenemia (fibrinogen < 150 mg/dL)	1.45 g/L
Hemophagocytosis in bone marrow	Positive
Ferritin > 500 ng/ml	2400 ng/ml
Elevated soluble CD25	2367 U/L
NK cell activity	12.71%
CNS symptoms	Seizure

HLH, hemophagocytic lymphohistiocytosis; CNS: central nervous system.

**Table 2:** Cytokine levels of the patient.

Cytokine	Cytokine level (pg/ml) on days relative to emapalumab initiation		
	D-7	D+14	D+46
Interleukin-1β	≤ 2.3 pg/ml	≤ 2.3 pg/ml	≤ 2.3 pg/ml
Interleukin-2	≤ 2.3 pg/ml	≤ 2.3 pg/ml	≤ 2.3 pg/ml
Interleukin-4	≤ 2.3 pg/ml	≤ 2.3 pg/ml	≤ 2.3 pg/ml
Interleukin-5	≤ 2.3 pg/ml	≤ 2.3 pg/ml	≤ 2.3 pg/ml
Interleukin-6	39.03 pg/ml	2.73 pg/ml	≤ 2.3 pg/ml
Interleukin-8	3.99 pg/ml	5.63 pg/ml	≤ 2.3 pg/ml
Interleukin-10	8.96 pg/ml	≤ 2.3 pg/ml	≤ 2.3 pg/ml
IFN-γ	46.82 pg/ml	≤ 2.3 pg/ml	≤ 2.3 pg/ml
Tumor necrosis factor-α	≤ 2.3 pg/ml	≤ 2.3 pg/ml	≤ 2.3 pg/ml

**Figure 1:**
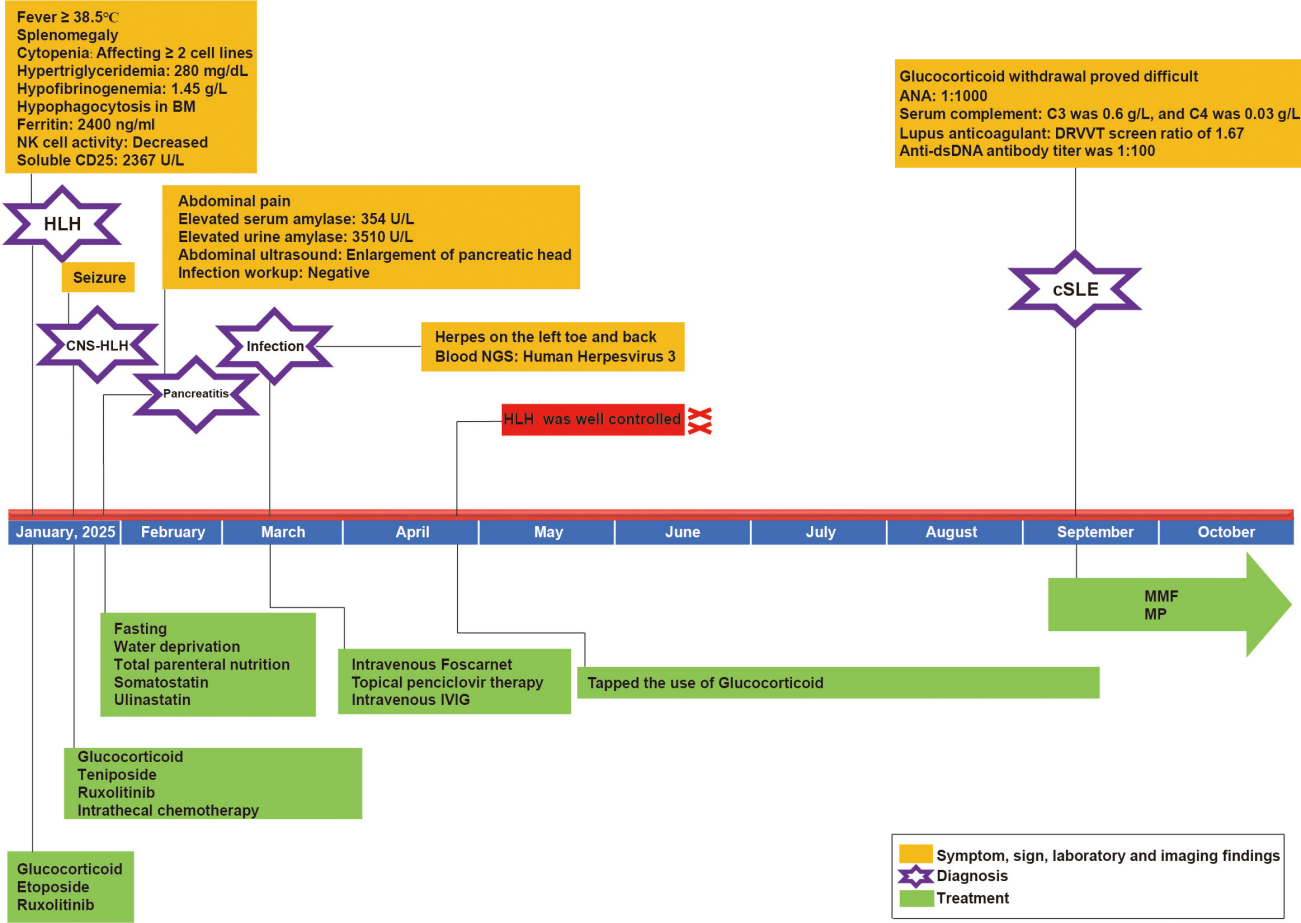
Clinical timeline and treatment of a pediatric patient with SLE-induced HLH. SLE, systemic lupus erythematosus; HLH, hemophagocytic lymphohistiocytosis; BM, bone marrow; NGS, next-generation sequencing; ANA, antinuclear antibody; CNS, central nervous system; IVIG, intravenous immunoglobulin; MP, methylprednisolone; MMF, mycophenolate mofetil; DRVVT, diluted Russell’s viper venom time; cSLE, childhood-onset SLE.

Since the patient met all the HLH criteria, she was initially treated with the HLH-94/HLH-2004 therapeutic protocol (**Figure 1**), which consisted of 150 mg/m² etoposide (VP–16) twice weekly during week 1 and methylprednisolone at a dosage of 10 mg/kg/day from January 16 to 18, 2025, which was tapered to 5 mg/kg/day from January 19 to 21, 2025; unfortunately, the disease progressed after one week of treatment with this protocol. On January 24, 2025, the patient reported abdominal pain. Laboratory tests revealed that her serum amylase was elevated to 354 U/L (normal range: 35–135), while her urine amylase was elevated to 3510 U/L (normal range: 0–460), and abdominal ultrasound suggested pancreatic head enlargement. An infectious etiology was excluded, and pancreatitis secondary to HLH was considered. Management included fasting and water deprivation, total parenteral nutrition, a continuous somatostatin infusion, and an intravenous ulinastatin infusion. On January 25, 2025, a lumbar puncture was performed because of the patient’s frequent neuropsychiatric symptoms, including a rapidly and progressively altered mental status, seizures, and intracranial hypertension, which differed from more common symptoms such as headache, cognitive dysfunction, psychotic symptoms, aseptic meningitis and focal neurological deficits. Given that the patient had received high-dose glucocorticoid therapy two weeks prior to admission without developing any neurological symptoms, a drug-related cause for these adverse neurological effects was excluded. Cerebrospinal fluid (CSF) analysis revealed a leukocyte count of 8 × 10^6^/L and an elevated protein concentration (0.6 g/L) (reference range 0.15–0.45 g/L). CSF-pathogen metagenomic next-generation sequencing (mNGS) was negative for bacteria, fungi, viruses, and rare opportunistic pathogens. Electroencephalography (EEG) revealed diffuse moderate abnormal background activity without focal epileptic discharge. Serial re-examinations of the CSF revealed persistently elevated protein levels with mild lymphocytic pleocytosis, lacking the dynamic changes typical of infectious or metabolic encephalopathy. Cranial magnetic resonance imaging revealed diffuse white matter lesions, meningeal enhancement, and multifocal parenchymal abnormalities, characteristic imaging manifestations of central nervous system hemophagocytic lymphohistiocytosis (CNS-HLH) (**Figure 1**; **Table 3**).

**Table 3:** HLH treatment protocol.

Day (Date)	Chemotherapy drugs and dosage per body surface area (m^2^)				Bone marrow biopsy	Intrathecal chemotherapy (IT chemo)	Note
	Glucocorticoid	Etoposide	Ruxolitinib	Emapalumab			
	Methylprednisolone						
2025-01-16	10 mg/kg		2.5 mg BID				
2025-01-17	10 mg/kg	100 mg/m^2^	2.5 mg BID			
2025-01-18	10 mg/kg		2.5 mg BID			IT
2025-01-19	5 mg/kg		2.5 mg BID				
2025-01-20	5 mg/kg	100 mg/m^2^	2.5 mg BID				CSF: leukocyte count 8 × 10^6^/L, protein concentration 0.6 g/L. EEG: moderate abnormal background activity without focal epileptic discharges. No changes on serial reexaminations. Head MRI: diffuse white matter lesions, meningeal enhancement, and multifocal parenchymal abnormalities. Diagnosis: CNS- HLH.
2025-01-21	5 mg/kg		2.5 mg BID			
	Dexamethasone		2.5 mg BID				
2025-01-22	10 mg/m^2^		2.5 mg BID	2 mg/kg			
2025-01-23	10 mg/m^2^	Teniposide	2.5 mg BID				
2025-01-24	10 mg/m^2^	50 mg/m^2^	2.5 mg BID				
2025-01-25	10 mg/m^2^		2.5 mg BID	2 mg/kg		IT	
2025-01-26	10 mg/m^2^		2.5 mg BID				
2025-01-27	10 mg/m^2^		2.5 mg BID				
2025-01-28	10 mg/m^2^		2.5 mg BID	2 mg/kg			
	Methylprednisolone						
2025-01-29	30 mg		2.5 mg BID				
2025-01-30	30 mg	50 mg/m^2^	2.5 mg BID				
2025-01-31	25 mg	50 mg/m^2^	2.5 mg BID				
2025-02-01	20 mg		2.5 mg BID				
2025-02-02	20 mg		2.5 mg BID	2 mg/kg			
2025-02-03	16 mg		2.5 mg BID				
2025-02-04	16 mg		2.5 mg BID				
2025-02-05	16 mg		2.5 mg BID	2 mg/kg			
2025-02-06	16 mg		2.5 mg BID				
2025-02-07	16 mg	50 mg/m^2^	2.5 mg BID				
2025-02-08	16 mg	50 mg/m^2^	2.5 mg BID				
2025-02-09	16 mg		2.5 mg BID	2 mg/kg			
2025-02-10	16 mg		2.5 mg BID				
2025-02-11	16 mg		2.5 mg BID				
2025-02-12	12 mg		2.5 mg BID	2 mg/kg			
2025-02-13	12 mg		2.5 mg BID				
2025-02-14	12 mg	50 mg/m^2^	2.5 mg BID				
2025-02-15	12 mg	50 mg/m^2^	2.5 mg BID				
2025-02-16	12 mg	50 mg/m^2^	2.5 mg BID				
2025-02-17	12 mg		2.5 mg BID				
2025-02-18	12 mg		2.5 mg BID				
2025-02-19	10 mg		2.5 mg BID	2 mg/kg			
2025-02-20	10 mg	50 mg/m^2^	2.5 mg BID				
2025-02-21	10 mg	50 mg/m^2^	2.5 mg BID				
2025-02-22	10 mg	50 mg/m^2^	2.5 mg BID				
2025-02-23	10 mg		2.5 mg BID				
2025-02-24	10 mg		2.5 mg BID				
2025-02-25	10 mg		2.5 mg BID	2 mg/kg			
2025-02-26	8 mg		2.5 mg BID				
2025-02-27	8 mg	50 mg/m^2^	2.5 mg BID				
2025-02-28	8 mg	50 mg/m^2^	2.5 mg BID				
2025-03-01	8 mg	50 mg/m^2^	2.5 mg BID				
2025-03-02	8 mg		2.5 mg BID				
2025-03-03	8 mg						
2025-03-04	8 mg						
2025-03-05	6 mg						
2025-03-06	6 mg						
2025-03-07	6 mg						
2025-03-08	10 mg						
2025-03-09	10 mg						
2025-03-10	10 mg						
2025-03-11	10 mg						
2025-03-12	10 mg						
2025-03-13	10 mg						
2025-03-14	8 mg						

HLH, hemophagocytic lymphohistiocytosis; CSF, cerebrospinal fluid; EEG, electroencephalogram; MRI, magnetic resonance imaging; IT, intrathecal.

Consequently, the treatment regimen was modified as follows. First, the patient received dexamethasone (10 mg/m^2^/day) from January 22 to January 28, 2025. From January 29 to February 2, 2025, the methylprednisolone dosage was tapered from 30 mg daily to 20 mg daily, after which it was adjusted further to 16 mg daily from February 3, 2025, to February 11, 2025. The regimen was continued at 12 mg daily from February 12 to February 18, 2025, followed by a further reduction to 10 mg daily from February 19 to February 25, 2025. The dosage was further tapered to 8 mg daily from February 26 to March 4, 2025, and subsequently to 6 mg daily from March 5 to March 7, 2025. During the steroid tapering period, the patient experienced a reduction in peripheral blood cell counts. The methylprednisolone dosage was therefore increased from 6 mg daily to 10 mg daily starting on March 7 and then tapered again to 8 mg daily from March 14 onward. Teniposide was selected instead of the structurally similar etoposide, as numerous studies have documented key differences that would favor the former in this case. Specifically, teniposide is preferred over etoposide for treating CNS hemophagocytic encephalopathy mainly because of its higher lipophilicity and superior blood–brain barrier penetration, which results in elevated intracerebral drug concentrations and better therapeutic effects on intracranial lesions. Moreover, teniposide is more effective at controlling central nervous system inflammatory infiltration, whereas etoposide does not distribute sufficiently throughout the brain and is more suitable for treating systemic tumors and conventional hematological malignancies (4, 5). Given these findings and our previous single-center clinical experience, we chose to use teniposide to better ensure the patient’s survival. Teniposide was administered at 50 mg/m^2^ per week on January 24, 30, and 31 and February 7 and 8, 2025; at 50 mg/m^2^ on February 14, 15, 16, 20, 21, 22, 27, and 28, 2025; and then again at 50 mg/m^2^ per week on March 1, 2025. The patient received 2.5 mg of ruxolitinib twice daily for 2 months. In recent reports, the initial dosage of emapalumab was 1 mg per kilogram of body weight every 3 days, with early dosage adjustments guided by both clinical and pharmacokinetic assessments. Subsequent protocol amendments simplified the dosing strategy to rely solely on predefined clinical and laboratory criteria, allowing escalation from the starting dose of 1 mg/kg to 3 mg/kg, 6 mg/kg, and a maximum of 10 mg/kg according to disease activity responses (6). However, owing to financial constraints and limited drug availability at that time, the standard dosing schedule was not followed for this patient; instead, the patient received emapalumab 2 mg/kg once daily on January 22, 25 and 28 and February 2, 5, 9, 12, 19, and 25 (**Table 3**). Dexamethasone and methotrexate were administered intrathecally for disease control. During immunosuppressive therapy targeting HLH, quantitative PCR was performed weekly to monitor the patient for herpesviruses, including Epstein–Barr virus (EBV) and cytomegalovirus (CMV); however, the patient was not placed under routine surveillance for human herpesvirus 3 (HHV-3). Unfortunately, despite initial negative screening, viremia was detected on March 1, 2025, necessitating management with antiviral prophylaxis with acyclovir. The patient developed herpetic lesions on her left toes and back. Blood NGS confirmed infection with HHV-3, with a peripheral blood viral load reaching 2.95 × 10^6^ copies/mL, for a relative abundance of 92.22%. The antiviral regimen was subsequently switched to foscarnet and topical penciclovir treatments and was supplemented by intravenous immunoglobulin (IVIG) therapy. After two months of these treatments, the patient’s facial rash gradually subsided, no new rashes appeared, and the oral mucosal lesions on the upper jaw regressed. The levels of ferritin and all cytokines were normal. Triglyceride (1.5 mmol/L) and ferritin (805 μg/L) concentrations were lower than the previous values; specifically, on March 5, 2025, their values were 1.72 mmol/L and 1428 μg/L, respectively. The oral dosage of methylprednisolone was gradually tapered. By April 24, 2025. However, during hormone reduction, multiple follow-up routine blood tests revealed significant decreases in WBC and PLT counts and significant increases in cholesterol and triglyceride levels. Bone marrow aspiration consistently indicated the presence of hemophagocytic cells. During the 4-month period of repeated glucocorticoid dosage adjustment, the peripheral blood parameters improved correspondingly as the steroid dosage was increased. On September 14, owing to the challenge of tapering the glucocorticoids, we screened the patient again for rheumatic and autoimmune diseases. The relevant tests were conducted in accordance with the 2019 EULAR/ACR criteria for childhood-onset systemic lupus erythematosus (cSLE). The entry criterion was an ANA titer of 1:1000 (defined as ≥ 1:80). The serum complement C3 concentration was 0.6 g/L, which was significantly below the normal reference interval of 0.9–1.8 g/L, and the complement C4 concentration was 0.03 g/L, which was below the normal range of 0.1–0.4 g/L. The results of lupus anticoagulant testing were positive, with a diluted Russell’s viper venom time screen ratio of 1.67, which was above the normal cutoff of < 1.2. The anti-dsDNA antibody titer was 1:100, which was significantly higher than the normal upper limit of 1:10. In the early stage of the disease, the patient presented with a fever ≥ 38.3 °C, oral ulcers, and subacute cutaneous or discoid lupus as well as delirium, psychosis, and seizures. Laboratory tests revealed leukopenia (WBC count 600/mm³, criterion < 4,000/mm³) and thrombocytopenia (PLT count 62,000/mm³, criterion < 100,000/mm³). The patient’s total diagnostic score was 27 points (threshold ≥ 10 points); on the basis of these findings as well as the patient’s medical history, the final diagnosis was hemophagocytic syndrome induced by cSLE (**Figure 1**). The specific scores are shown in **Table 4**.

**Table 4:** Patient characteristics at diagnosis of cSLE.

2019 EULAR/ACR cSLE criteria	Case findings	Score
Entry criterion ANA at a titer of ≥1:80	1:1000	
Fever ≥ 38.3 °C	Positive	2
Mucocutaneous symptoms Nonscarring alopecia Oral ulcers (2 points if present) Subacute cutaneous or discoid lupus Acute cutaneous lupus	Negative Positive Positive Negative	0 2 4 0
Musculoskeletal Joint involvement	Negative	0
Neuropsychiatric symptoms Delirium Psychosis Seizure	Positive Positive Positive	2 3 5
Serosal symptoms Pleural or pericardial effusion Acute pericarditis	Negative Negative	0 0
Hematologic symptoms Leukopenia (white blood cell count < 4,000/mm³) Thrombocytopenia (platelet count < 100,000/mm³) Autoimmune hemolysis	600/mm³ 62×10^9^/L	3 4 0
Renal Proteinuria > 0.5 g/24 h Renal biopsy class II or V lupus nephritis Renal biopsy class III or IV lupus nephritis	Negative Negative Negative	0 0 0
Antiphospholipid antibodies Lupus anticoagulant DRVVT screen ratio (< 1.2) Anti-cardiolipin IgA Ab (APLU/ml) Anti-cardiolipin IgG Ab (GPLU/ml) Anti-cardiolipin IgM Ab (MPLU/ml)	1.67 Negative Negative Negative	2 0
Complement proteins Low C3 points (0.9-1.8 g/L) Low C4 points (0.1–04 g/L)	C3 0.6 C4 0.03	4
SLE-specific antibodies Anti-dsDNA antibody Anti-Smith antibody	1:100 Negative	6 0

cSLE, childhood-onset systemic lupus erythematosus; DRVVT, diluted Russell’s viper venom time.

We adjusted the dosage of methylprednisolone to 12 mg/d and added 500 mg/d oral mycophenolate mofetil tablets. At the follow-up visit on November 22, 2025, the ANA level had decreased to 1:160; the anti-dsDNA antibody level had decreased to 1:10, and laboratory results indicated a WBC count of 4.2 × 10^9^/L, a PLT count of 135 × 10^9^/L, a ferritin concentration of 130.30 µg/L, a total cholesterol concentration of 5.53 mmol/l, and a triglyceride concentration of 1.27 mmol/L. After a follow-up period of more than one year, ending on May 10, 2026, the clinical condition of the pediatric patient with cSLE remained stable. The patient was maintained on oral mycophenolate mofetil (MMF) at 500 mg and methylprednisolone at 5 mg daily and had no recurrent CNS manifestations throughout the follow-up period.

As of the writing of this report, all clinical indicators have improved, and the patient is undergoing regular follow-up.

## Discussion

HLH is an acute and life-threatening systemic inflammatory disease characterized by uncontrolled lymphocyte and macrophage activation and excessive cytokine secretion (3). The HLH-2004 criteria remain the current standard for diagnosing the disease. HLH can be diagnosed if either of the following two criteria is met: first, molecular diagnosis via identification of pathological mutations in known HLH-related pathogenic genes, such as *PRF1*, *UNC13D*, *STX11*, *STXBP2*, *Rab27a*, *LYST*, *SH2D1A*, *BIRC4*, *ITK*, *AP3β1*, *MAGT1*, or *CD27*; and second, 5 or more of the following 8 indicators: (1) Fever: body temperature > 38.5 °C for more than 7 days. (2) Splenomegaly. (3) Reductions in hematopoietic cells involving two or three peripheral blood lineages. This includes a hemoglobin concentration < 90 g/L (or Hb concentration < 100 g/L for infants under 4 weeks old), a PLT count < 100 × 10^9^/L, and a neutrophil count < 1.0 × 10^9^/L, with reduced bone marrow function excluded as a possible cause. (4) Hypertriglyceridemia and/or hypofibrinogenemia: triglyceride concentration > 3 mmol/L or more than 3 standard deviations above the age-specific mean and fibrinogen levels < 1.5 g/L or more than 3 standard deviations below the age-specific mean. (5) Detection of hemophagocytic phenomena in the bone marrow, spleen, liver or lymph nodes. (6) Decreased or absent NK cell activity. (7) Elevated serum ferritin concentration (≥ 500 μg/L). (8) Elevated sCD25 (soluble interleukin-2 receptor) levels (7).

HLH is divided into pHLH and sHLH; the former is an autosomal or sex-linked recessive genetic disorder, whereas the latter is an inflammatory disease characterized by excessive immune system activation of various etiologies, such as infections, tumors, and rheumatic diseases. Forms of HLH associated with rheumatologic diseases, most commonly systemic juvenile idiopathic arthritis and SLE, are termed MAS (8).

However, this classification system remains a subject of ongoing debate, as pHLH and sHLH share overlapping genetic features; notably, some genes may contribute to the development of sHLH (9). In the case presented here, we conducted comprehensive genetic testing but identified no pathogenic variants. We also tested the patient for relevant pathogens and conducted rheumatologic and immunologic evaluations, but no definitive secondary triggers were identified. There was no family history of similar disorders in the patient. Therefore, the cause of HLH was initially undetermined. We also hypothesized that the patient’s clinical presentation might have been masked by the steroid therapy administered prior to hospitalization. However, throughout the subsequent management, we continued to investigate the etiology of her condition and ultimately identified a case of hemophagocytic syndrome caused by cSLE. In cases of hemophagocytic syndrome of unknown origin, clinicians must continue to search for the underlying etiology rather than ceasing investigation prematurely.

Currently, the HLH-94 protocol remains the standard treatment for HLH. In this protocol, a combination of dexamethasone and etoposide is employed to achieve disease control, followed by allogeneic HSCT, which is the only definitive treatment for pHLH (10, 11). Although approximately 80% of patients respond to the initial therapies and can subsequently undergo HSCT, fewer than half achieve complete remission. Most deaths occurring prior to HSCT appear to result from an inability to control disease activity (10). Moreover, long-term treatment with these drugs is associated with substantial toxicity, including severe bone marrow toxicity, an increased risk of opportunistic infections, obesity, diabetes, hypertension, and other adverse reactions (12). Accordingly, it is necessary to identify more promising and tolerable therapeutic approaches. Consequently, in the current patient, teniposide was replaced with etoposide. Both drugs are podophyllotoxin-derived semisynthetic antitumor agents that act as topoisomerase II inhibitors. Compared with etoposide, however, teniposide is more lipophilic and permeates the blood–brain barrier more effectively; moreover, teniposide can more readily enter into lymphoid tissue, brain tissue and tumor tissue and can better target tissues and infiltrate tumors and intracranial lesions. In contrast, the penetrability of etoposide into the brain is low; thus, etoposide has limited effects on intracranial inflammation and aberrant immune cell clearance. Teniposide is therefore preferred for hematological malignancies accompanied by central nervous system involvement, manifesting with symptoms such as convulsions, altered consciousness, intracranial hypertension, abnormal cerebrospinal fluid results and radiologically confirmed intracranial infiltration. While etoposide has favorable systemic therapeutic effects, its insufficient blood–brain barrier permeability results in slow control, and treated central lesions have a high risk of recurrence. During the management of this case, guided by our institutional clinical experience, we administered teniposide in lieu of etoposide. Long-term follow-up has confirmed sustained neurological improvement in this patient (13, 14).

In recent years, targeted therapy has been increasingly applied in the management of hematological diseases. Its potential clinical benefits include favorable clinical outcomes, reduced medication doses, and fewer side effects. Cytokine storms play important roles in the occurrence of HLH. Moreover, increasing evidence has demonstrated that IFN-γ plays a key role in the pathogenesis of cytokine storms. The levels of IFN-γ and chemokine ligand 9 (CXCL9)—which is induced by IFN-γ—have been found to be significantly greater in patients with HLH related to rheumatic diseases than in those with active systemic juvenile idiopathic arthritis without HLH. These levels returned to normal after successful HLH treatment, suggesting that IFN-γ could be a feasible target for controlling excessive pathological inflammation (15). In this case, the patient’s IFN-γ level was markedly elevated upon presentation.

Emapalumab is a fully human IgG1 monoclonal antibody that neutralizes both free and receptor-bound IFN-γ. The pharmacological characteristics of this drug, which exhibit a nonlinear relationship with the drug dose, include targeted-mediated drug distribution (TMDD) (11). In November 2018, emapalumab was approved by the FDA for treating children and adults with pHLH who have refractory, relapsed, or progressive disease or who are intolerant to conventional HLH treatments. Concurrently, prevention of viral infection, *Pneumocystis jirovecii* and fungal infections is necessary (2). In this case, acyclovir was used prophylactically to prevent viral infection, caspofungin was used to prevent fungal infections, and compound sulfamethoxazole tablets were used to prevent *Pneumocystis carinii* infection. However, the patient still developed HHV-3 infection during treatment; consequently, the antiviral therapy was switched to foscarnet and intermittent IVIG infusion, and the patient gradually improved.

Numerous studies have demonstrated the therapeutic efficacy of monoclonal antibodies. A real-world study in the United States that included cases of HLH treated with monoclonal antibodies across 33 hospitals between November 20, 2018, and October 31, 2021 revealed that multiple laboratory parameters related to HLH rapidly returned to normal following emapalumab treatment, indicating a positive response. The probability of a treatment response was negatively correlated with the serum CXCL9 level. This chemokine is induced only by IFN-γ and thus serves as a biomarker for IFN-γ activity. In that study, CXCL9 levels decreased following emapalumab treatment. Among patients receiving emapalumab, more than 90% were deemed eligible for HSCT, with a pretransplant survival rate of 90%. Approximately 74% of the patients who were deemed suitable for transplantation subsequently underwent HSCT. The 12-month survival rate after HSCT was 73.7%, and the survival rate of the patients who underwent HSCT at the end of the study period was 74.2% (16). The JAK-STAT pathway is involved in many signaling transduction processes that are triggered when cytokines such as IFN-γ, IL-2, and IL-6 bind to specific receptors. Several studies have confirmed that the JAK1/2 inhibitor ruxolitinib significantly alleviated the manifestations of HLH and improved survival rates while limiting the expansion of CD8+ T cells and the production of proinflammatory cytokines. Given the significant increase in IL-6 levels in the patient in this case report, ruxolitinib was administered. Reports have indicated that the combination of emapalumab and ruxolitinib demonstrates favorable outcomes in treating refractory HLH caused by EBV (17).

The patient showed a favorable response to the treatment. Within two weeks, the relevant laboratory parameters had gradually decreased. However, three weeks later, the patient developed CNS-HLH, which mainly presented as convulsions. Therefore, methylprednisolone was switched to dexamethasone because of its superior penetration of the blood–brain barrier. However, HLH is a complex condition, and treatment protocols must be continuously tailored to the patient’s clinical status. Studies indicate that approximately one-third of patients present with neurological symptoms, which can lead to irreversible damage. Additionally, relapse is common (18), underscoring the need for vigilance. Emapalumab has exhibited promising efficacy in the treatment of CNS-HLH.

IFN-γ plays an important role in the development of HLH. However, the cytokine storm that occurs in this disease is highly complex. Other proinflammatory factors are also involved in the pathogenesis of HLH; these include several cytokines, such as IL-2, TNF-a, IL-6, IL-18, and IL-33 (19). Some drugs targeting these cytokines are currently in clinical trials or are being used in clinical practice; examples include alemtuzumab, which targets CD52, and tocilizumab, which targets IL-6R.

In pediatric patients, emapalumab is typically used as a transitional therapy for HSCT. Further research is needed to determine whether complete remission can be induced in treated patients without the use of HSCT. In China, emapalumab was approved for use by the National Medical Products Administration in March 2022. Therefore, owing to the relatively recency of its approval and the high cost of emapalumab in China, its use in HLH patients remains limited, and related research remains scarce. By reporting this case, therefore, we hope to contribute to the literature on the use of emapalumab for HLH in China and worldwide. Given that this report is limited to a single case, further confirmation with a large-sample study is needed.

In conclusion, we implemented a combined therapeutic approach including low-dose ruxolitinib, emapalumab, teniposide and dexamethasone in a patient with SLE-associated CNS-HLH. This multimodal intervention was clinically feasible, and its efficacy may be related to the amelioration of her clinical manifestations. After long-term clinical follow-up, the patient has achieved sustained clinical remission, characterized by resolved HLH activity, well-stabilized SLE disease activity, and complete recovery from neurological manifestations. Owing to the concomitant administration of multiple supportive and targeted interventions, the observed clinical improvement cannot be definitively attributed to this regimen alone. Nevertheless, the results of this single-case experience suggests that such a combination strategy may be a promising therapeutic approach for patients with severe, refractory SLE-associated HLH with CNS involvement.

## Data Availability

The original contributions presented in the study are included in the article/supplementary material. Further inquiries can be directed to the corresponding authors.
